# Children with intellectual disabilities: Support in inclusive practice

**DOI:** 10.1192/j.eurpsy.2021.1033

**Published:** 2021-08-13

**Authors:** Y. Afanasyeva, M. Bratkova, Y. Sidneva

**Affiliations:** 1 Institute Of Special Education And Psychology Institute Of System Projects Institute Of Lifelong Learning Directorate Of Educational Programs Institute Of Education Content, Methods And Technology, Moscow City University, Moscow, Russian Federation; 2 Department Of Rehabilitation, Clinical and Research Institute of Emergency Pediatric Surgery and Trauma (CRIEPST), Moscow, Russian Federation; 3 Psychiatric Research Group, N.N.Burdenko National Medical Research Center of Neurosurgery, Moscow, Russian Federation

**Keywords:** children with intellectual disabilities, inclusive education, learning difficulties, behavior disorder

## Abstract

**Introduction:**

The inclusion process allows children with special educational needs to be included in a normative environment. A large group consists of children with intellectual disabilities and behavioral disorders, hey need medical and pedagogical rehabilitation due to their low learning ability, neurotic disorders, and mental distortion.

**Objectives:**

Study of psychophysical characteristics of children with intellectual disability and behavioral disorders.

**Methods:**

140 children with intellectual disabilities who have impairments in the neuro-psychological sphere (2017-2020 г.г.). Methods: medical and pedagogical, observation, examination, assessment.

**Results:**

Variants of the child’s psychophysical development: Option 1. (75%): children with a predominance of violations in behavior, emotional and volitional sphere. There is aggressiveness, inconsistency and impulsiveness of actions, lack of distance with an adult, and difficulties in complying with accepted norms and rules. Option 2 (25%): children with the following manifestations: timidity, tearfulness, distrust, fears, lack of initiative. All children have difficulty sleeping, eating disorders, and frequent psychosomatic illnesses.

**Conclusions:**

Children with intellectual disabilities in an inclusive practice need comprehensive assistance, taking into account different variants of their psychophysical characteristics. The studied children were found to have neurotic and neurosis-like disorders, as well as pathological personality development. All children have: low performance, lability of the nervous system, lack of voluntary regulation, impaired activity, learning difficulties. There are behavioral and mental disorders that require medical, psychological and pedagogical rehabilitation.

